# Strategies for improving fengycin production: a review

**DOI:** 10.1186/s12934-024-02425-x

**Published:** 2024-05-22

**Authors:** Ying Yin, Xin Wang, Pengsheng Zhang, Pan Wang, Jianping Wen

**Affiliations:** 1 Key Laboratory of Systems Bioengineering (Ministry of Education), Tianjin University, Tianjin, 300072 P. R. China; 2 SynBio Research Platform, Collaborative Innovation Center of Chemical Science and Engineering (Tianjin), School of Chemical Engineering and Technology, Tianjin University, Tianjin, China; 3 Frontiers Science Center for Synthetic Biology (Ministry of Education), Tianjin University, Tianjin, China; 4 Coll Biol & Pharmaceut Sci, China Three Gorges Univ, Yichang, 443002 P. R. China; 5 Department of Nuclear Medicine, The First Hospital of Shanxi Medical University, Collaborative Innovation Center of Molecular Imaging Precision Medical, Shanxi Medical University, Taiyuan, 030001 China

**Keywords:** Fengycin, Genetic engineering, Lipopeptide, NRPS, Optimization

## Abstract

Fengycin is an important member of the lipopeptide family with a wide range of applications in the agricultural, food, medical and cosmetic industries. However, its commercial application is severely hindered by low productivity and high cost. Therefore, numerous studies have been devoted to improving the production of fengycin. We summarize these studies in this review with the aim of providing a reference and guidance for future researchers. This review begins with an overview of the synthesis mechanism of fengycin via the non-ribosomal peptide synthetases (NRPS), and then delves into the strategies for improving the fengycin production in recent years. These strategies mainly include fermentation optimization and metabolic engineering, and the metabolic engineering encompasses enhancement of precursor supply, application of regulatory factors, promoter engineering, and application of genome-engineering (genome shuffling and genome-scale metabolic network model). Finally, we conclude this review with a prospect of fengycin production.

## Graphical abstract

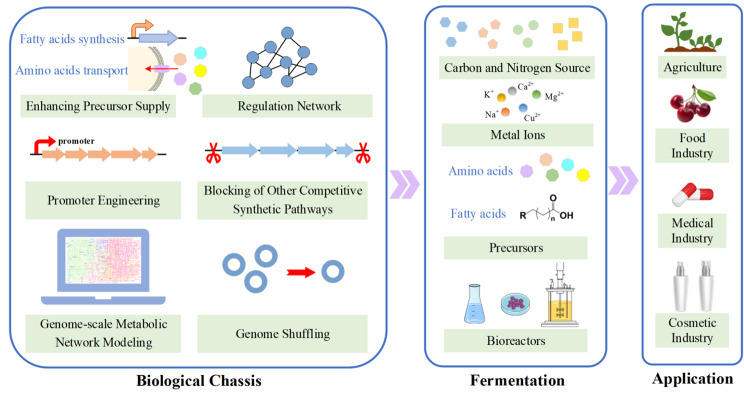

## Introduction

With an increasing emphasis on environmentally-friendly and sustainable development, biosurfactants are receiving more and more attention due to their advantages of low toxicity, high biodegradability, high environmental compatibility, and potential to be produced from renewable raw materials [1, 2]. As a class of biosurfactants, lipopeptides show great potential as biocontrol agents and pharmaceutical drugs, rendering them candidates for addressing microbial resistance [3, 4]. Polymyxins are the most well-known lipopeptides, with polymyxin B and polymyxin E (also known as colistin) having been used in clinical practice [4, 5]. Daptomycin has been approved by the Food and Drug Administration (FDA) for the treatment of infections caused by Gram-positive bacteria, with the market name of Cubicin [4]. In addition, since the FDA approved cilofungin, micafungin, and anidulafungin for the treatment of fungal infections in 2001, 2005, and 2006, respectively, more and more fungi lipopeptides and their chemically synthesized derivatives have been developed into commercial products or subjected to clinical trials, including caspofungin, rezafungin, emodepside, fusafungine and destruxins [3, 6]. Consequently, it is of great importance to undertake further investigation of lipopeptides.

Fengycin, surfactin and iturin A are the main lipopeptides produced by *Bacillus subtilis* [7]. Each lipopeptide has unique properties due to its specific amino acid sequences and variable fatty acid chain lengths [8]. Fengycin was initially discovered in the fermentation broth of *B. subtilis* F-29-3 and it was named fengycin A and fengycin B based on the difference at the sixth amino acid position [9]. Subsequently, fengycin S and fengycin C were found in the fermentation broth of *Bacillus amyloliquefaciens* LSC04 and *B. subtilis* EA-CB0015, respectively [10, 11]. In addition, plipastatin A and plipastatin B, which were extracted from the fermentation broth of *Bacillus cereus* BMG302-fF67 [12], also belong to the fengycin family.

Fengycin exhibits a more pronounced antagonistic effect on filamentous fungi compared with surfactin and iturin A, so it can be used as a treatment for various plant diseases without the negative impacts of chemical pesticides [13, 14]. Fengycin works by acting on the phospholipid bilayer of the cell membrane, causing structural damage and altering its permeability, which can result in the leakage of cellular contents and ultimately lead to cell death [15, 16]. Additionally, fengycin can induce the systemic resistance in plants through its action on root cell membranes [13]. The inhibitory mechanisms of fengycin give it the ability to prevent resistance, making treatment with fengycin advantageous. Fengycin also has potential applications in the food industry. It has been proved that fengycin exhibits a significant inhibitory effect on *Rhizopus stolonifer* and *Alternaria alternata*, which are responsible for postharvest soft rot [17, 18]. Therefore, it can be used for food preservation of fruits, such as cherries, apples and peaches. Besides, its activity against *Shewanella putrefaciens* makes it a promising natural food preservative for aquatic products [19]. Moreover, fengycin also has potential applications in the cosmetic industry [20, 21], environmental remediation, and petroleum development [22, 23]. Surprisingly, its anti-cancer activity in inhibiting the growth of human lung cancer cells [24], human colon cancer cells [25], and human leukemia cells [26] has also been revealed. Fengycin has also been reported to promote the topically treatment of localized dermatomycoses [27, 28].

Due to the high application value of fengycin, it is essential to increase its production. Currently, microbial production remains the primary method for producing fengycin due to the severe limitations in chemical synthesis of lipopeptides caused by their complex structure, and a few strains, mainly *Bacillus* spp., have been identified with the ability to synthesize fengycin. This review provides a brief introduction to the mechanism of fengycin synthesis from the perspective of non-ribosomal peptide synthetase (NRPS) and focuses on the strategies to improve fengycin production in current studies, particularly metabolic engineering strategies.

## Synthesis mechanism of fengycin

**Fig. 1:**
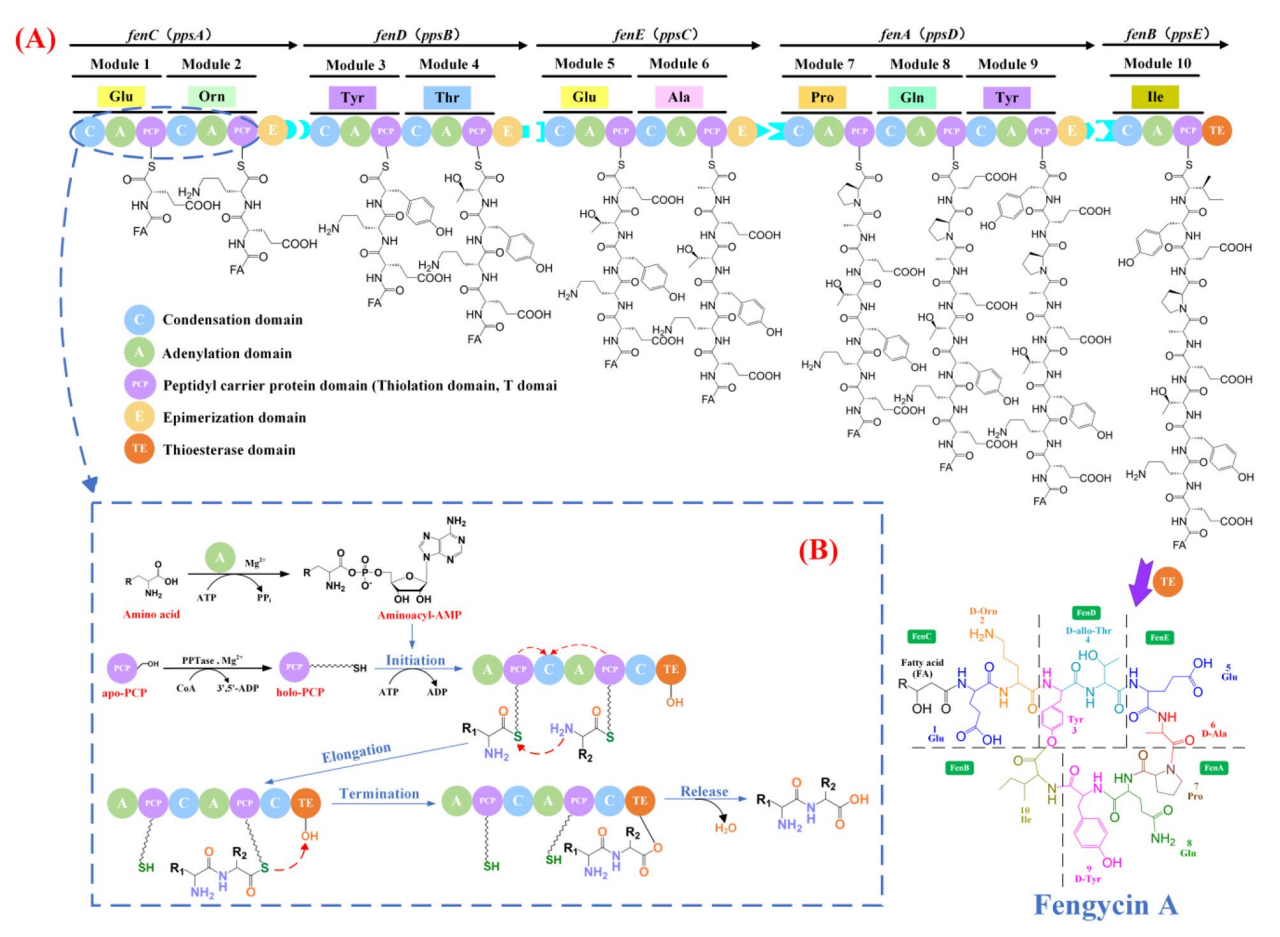
A schematic diagram of fengycin A synthesis. (**A**) The process of peptide chain elongation catalyzed by the synthases encoded by the *fen* (*pps*) gene cluster. (**B**) The mechanism of biosynthesis of NRPS with adenylation domain, condensation domain, peptidyl carrier protein domain and thioesterase domain

In a similar manner to other important non-ribosomal peptide (NRP) natural products, such as vancomycin, bleomycin, cyclosporine, and surfactin, the synthesis of fengycin is accomplished via NRPS [29, 30]. The NRPS recognizes, activates and links amino acids in a specific order to synthesize peptides via the multicarrier thiotemplate mechanism. There are three mainly basic domains involved in this process: the adenylation domain (A domain), the condensation domain (C domain), and the peptidyl carrier proteins domain (PCP domain, also known as thiolation domain and T domain) (Fig. 1B) [31–34]. In addition, NRPS also contains epimerization domain (E domain), which catalyzes the conversion of L-amino acids to D-amino acids [35, 36].

Fengycin A is composed of L-Glu, D-Orn, L-Tyr, D-allo-Thr, L-Glu, D-Ala, L-Pro, L-Gln, D-Tyr and L-Ile. These amino acids are activated and assembled in a specific order by FenC, FenD, FenE, FenA and FenB, which are encoded by *fenC*, *fenD*, *fenE*, *fenA* and *fenB* (*ppsA*, *ppsB*, *ppsC*, *ppsD* and *ppsE* in *B. subtilis 168*, respectively) (Fig. 1A). The first step of fengycin synthesis is catalyzed by FenC. FenC is responsible for the identification and assembly of L-Glu and L-Orn, ultimately forming a dipeptide, L-Glu-L-Orn [37]. Subsequently, the dipeptide L-Glu-L-Orn is translocated to FenD, during which L-Orn is racemized to D-Orn by the E domains of FenC, and this process continues from one peptide synthase to another until the elongating peptide chain reaches FenB [38]. The conversion of L-Thr, L-Ala and L-Tyr to D-allo-Thr, D-Ala and D-Tyr is also catalyzed by the E domains of FenD, FenE and FenA, respectively [37, 39–41]. Once the last amino acid is attached to the whole peptide chain, the TE domain will terminate the reaction, cyclize, and release the entire peptide chain, resulting in the formation of fengycin as the final product [38].

It is worth noting that in the most NRPS complexes, the modules are distributed over different NRPSs, which requires selective interaction and communication to synthesize defined peptide products. The coordinated interplay between pairs of donor and acceptor of communication-mediating (COM) domains is essential for the correct localization of the enzyme in multi-enzyme complexes as well as the selective translocation of intermediates between adjacent synthases [42]. Hahn et al. [43] proposed that a donor COM domain (COM^D^) located at the C terminus of an aminoacyl- or peptidyl-donating NRPS and an acceptor COM domain (COM^A^) located at the N terminus of the accepting partner NRPS form a matching (compatible) set, required for the proper intermolecular interaction between adjacent modules. Interestingly, Liu et al. [44] discovered that the point mutation $\text{COM}^{\text{D}}_{ppsB}$ (the donor domain of *ppsB*), the deletion of $\text{COM}^{\text{D}}_{ppsC}$, the replacement of $\text{COM}^{\text{D}}_{ppsD}$ with $\text{COM}^{\text{D}}_{ppsC}$, the replacement of $\text{COM}^{\text{D}}_{ppsB}$ with $\text{COM}^{\text{D}}_{ppsD}$, and the replacement of $\text{COM}^{\text{A}}_{ppsC}$ (the acceptor domain of *ppsC*) with $\text{COM}^{\text{A}}_{ppsB}$ can result in the formation of novel NRPS complex assembly lines *ppsA*/*ppsB*/*ppsE*, *ppsA*/*ppsB*/*ppsC*, *ppsA*/*ppsB*/*ppsC*/*ppsD*, *ppsA*/*ppsB*/*ppsC*/*ppsE*, and *ppsA*/*ppsB*/*ppsD*/*ppsE*, respectively, and in turn resulted in the formation of five new lipopeptides. Similar results can also be found in the biosynthesis of surfactin [42]. It represents a novel approach for the development of novel lipopeptides.

## Enhancing fengycin production through fermentation optimization

Optimizing fermentation conditions is pivotal for maximizing the production of target products. This subsection succinctly discusses the effects of carbon sources, nitrogen sources, metal ions, exogenous supply of precursors, and fermentation technologies on fengycin production.

### Effect of carbon source on fengycin production

The choice of carbon source significantly impacts fengycin yield. Although glucose is commonly used, alternative carbon sources, such as xylose [45–47], arabinose [48], fructose [49–52], mannitol [53], sucrose [54], glycerol [1, 55], and kitchen waste [56–58] have also been developed for fengycin production. Different carbon sources could affect fengycin production by affecting metabolic pathways, the expression of key genes, and the supply of energy and reductive power [46, 49–51]. It is worth noting that although the use of sustainable resources, such as glycerol, could promote a circular economy in industrial biotechnology, it may result in the increased production costs due to the difficulty of the post-processing process, which is an additional challenge that must be addressed alongside the low yields.

### Effect of nitrogen source on fengycin production

In addition to carbon source, nitrogen source also has a significant effect on fengycin production. Previous studies have demonstrated that combining nitrogen sources, such as glutamic acid with yeast extract, and urea with NH_4_HCO_3_, can result in higher fengycin yields [23, 59]. Furthermore, the carbon to nitrogen ratio is also crucial, with different ratios favoring different microbial strains. The optimal carbon to nitrogen ratio for lipopeptide production in *B. amyloliquefaciens* MEP218 is 10:1 using glucose and NH_4_NO_3_ [60], while Wei et al. [61] achieved the highest fengycin titer of 1220 mg/L with a carbon to nitrogen ratio of approximately 8. Since different strains may have different nitrogen source requirements, it is advisable to perform a case-by-case analysis of the selection.

### Effect of metal ions on fengycin production

Metal ions are involved in intracellular metabolic activities as activators of various enzymes. And it has been proved that K^+^, Na^+^ and Cu^2+^ can promote fengycin production [62, 63]. Cu^2+^ could increase fengycin titer by up-regulating the expression of *fenD* and *fenE* [63]. Besides, Mg^2+^ participates in fengycin synthesis as an activator of PNPase catalytic activity and a cofactor for Sfp [34, 64, 65]. In contrast, high concentrations of Ca^2+^ were found to be detrimental to the production of fengycin and surfactin by the regulation of two-component systems ResD/ResE, PhoP/PhoR, and DegU/DegS [62].

### Effect of exogenous supply of precursors on fengycin production

Fengycin consists of a chain of β-hydroxy fatty acids and 10 amino acid residues [45], thus the supply of fatty acids and amino acids is essential for fengycin production. It has been demonstrated that the addition of fatty acids (including myristic acid, pentadecanoic acid, heptadecanoic acid, nonadecanoic acid, and C16 fatty acid) [14, 61] and amino acids (such as glutamic acid, asparagine, serine, alanine, lysine, and ornithine) [54, 56, 66, 67] significantly enhanced the production of fengycin, in which the addition of alkanoic acid could up-regulate the transcription levels of synthetic genes *fenA*, *srfAA*, *ituD*, and fatty acid metabolism-related genes *fabI* and *fadB* [14], while addition of 10 g/L glutamic acid enhanced fengycin production mainly by up-regulating the expression of membrane transport systems [54]. Nevertheless, the addition of the identical amino acids may also result in different effects on fengycin production in different strains [54, 61, 66]. This variation may be attributed to differences in the distribution of metabolic fluxes in different strains, which in turn result in varying requirements for precursor amino acids.

### Advances in fermentation technology for fengycin production

In addition to the medium, the fermentation environment is also an important factor in fengycin production. It has been reported that the application of a bubble-free hollow fiber membrane bioreactor and solid-state fermentation could enhance the production of fengycin while reducing the negative effects of traditional aeration methods, such as the formation of excessive bubbles [68, 69]. Notably, in solid-state fermentation, carbon was directed towards the synthesis of lipopeptides rather than primary metabolites, making it a viable alternative to submerged fermentation for improving the efficiency and scalability of fengycin production [69]. In addition, cell immobilization has also been successfully applied in the fermentation of fengycin, resulting in higher productivity than that observed in free cell systems [23, 70–72]. Although new fermentation technologies can contribute to increased fengycin yields, there are still significant obstacles to be overcome before they can be applied on an industrial scale.

## Enhancing fengycin production through metabolic engineering

To meet with the market demand for fengycin, numerous studies have been devoted to increasing the production of fengycin through metabolic engineering approaches, including enhancement of precursor supply [46, 48, 54, 56, 57, 61, 73–75], application of regulatory factors [8, 45, 64, 75–84], promoter engineering [45, 48, 67, 75, 79, 85–87], and application of genome-engineering (genome shuffling [20, 88] and genome-scale metabolic network model [74]).

### Enhancement of precursor supply

#### Enhancing the supply of amino acids

As mentioned previously, exogenous supply of amino acids can effectively increase fengycin production [54, 56, 66, 67]. Concurrently, the utilization of exogenous amino acids requires the assistance of amino acid transporters and efficient expression of related proteins is crucial for fengycin synthesis. Gao et al. [73] discovered that overexpression of genes involved in the transport of proline, alanine, isoleucine and threonine can significantly increase fengycin yield. Additionally, they found that the combination of overexpression of *opuE*, which encodes the proline transporter protein, and addition of 8.0 g/L proline can increase fengycin production from 491.94 mg/L to 871.86 mg/L.

Compared with pure culture, microbial co-culture can modularize and disperse synthetic pathways into multiple strains, which can reduce the metabolic burden and stress on individual strains, minimizes interference between different metabolic pathways, and allows for the adjustment of metabolic fluxes between modules by altering the proportions of strains [57, 73]. Therefore, co-cultivating fengycin-producing strain with high amino acid-producing strain is a feasible method for improving fengycin production. *Corynebacterium glutamicum* is an industrial strain capable of producing various amino acids, making it an excellent choice for providing amino acids in a co-culture system [56]. Several studies have shown that co-cultivation of fengycin-producing strains with a series of *C. glutamicum*, which have high production of proline, serine, threonine, valine and isoleucine, resulted in significantly higher fengycin production due to the provision of sufficient amino acid precursors, compared with pure cultures [56, 57, 61, 73]. However, the introduction of an engineered *Saccharomyces cerevisiae*, which can hydrolyze the starch from food waste to provide carbon source, into the three-strain artificial consortia for fengycin production resulted in a decrease in lipopeptide yields [57]. This may be due to substrate competition and energy allocation in the co-culture system of the four strains. It’s worth noting that inoculation time and ratio of different strains, as well as culture medium used for co-cultivation are also significantly important for lipopeptides production [56, 61]. Therefore, although microbial co-culture has shown great potential for fengycin production, designing and optimizing microbial communities remains a major challenge.

#### Enhancing the supply of fatty acids

It has been proved that genes related to fatty acid synthesis exhibited significant changes in high fengycin-producing strains [49]. Several studies have identified that fatty acid supplementation is beneficial for the production of fengycin [46, 48, 54, 74, 75].

**Fig. 2:**
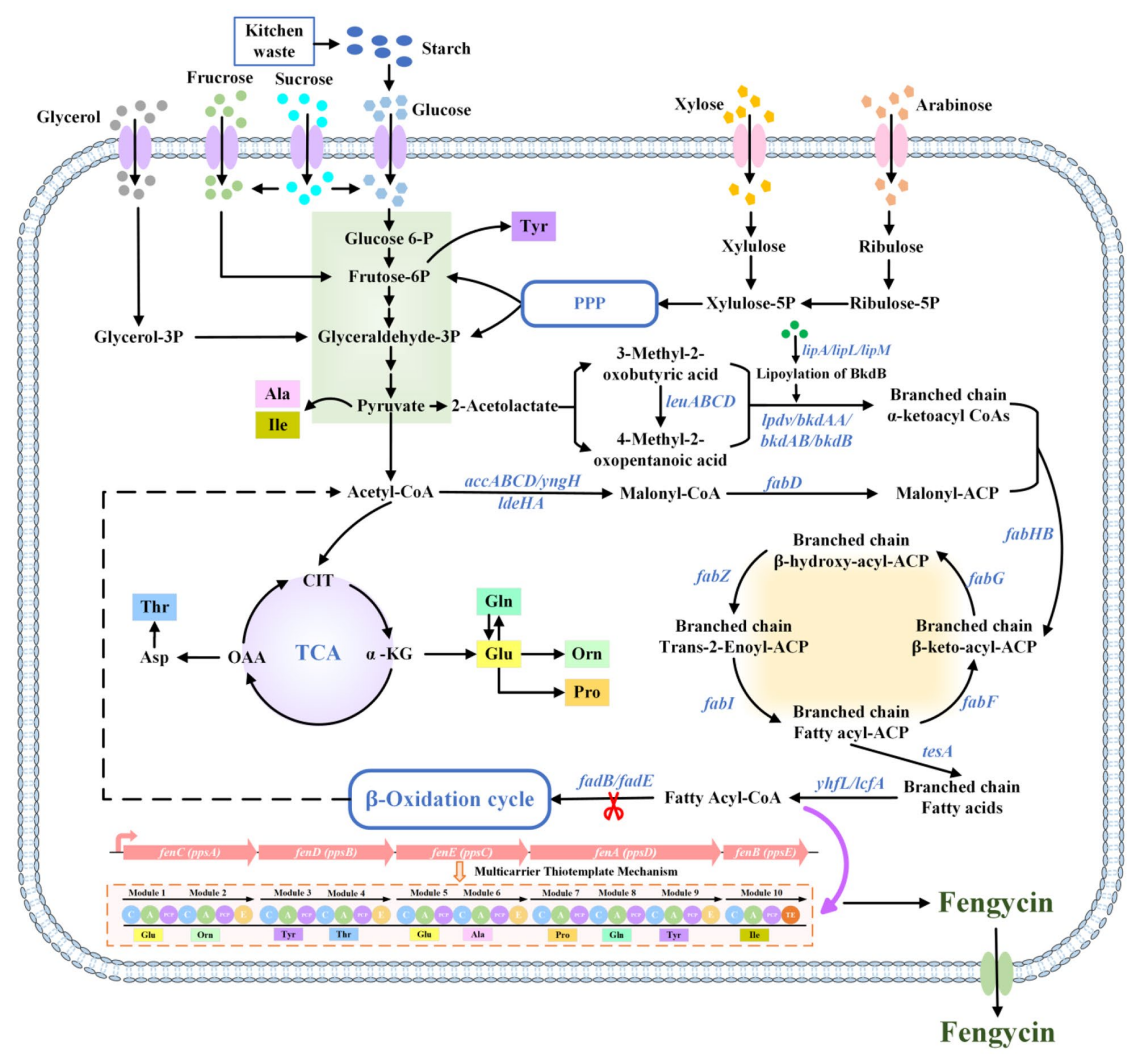
The metabolic pathway for fengycin synthesis. Xylulose-5P: xylulose-5-phosphate, Ribulose-5P: ribulose-5-phosphate; PPP: pentose phosphate pathway; Glucose-6P: glucose-6-phosphate; Frutose-6P: frutose-6-phosphate; Glyceraldehyde-3P: glyceraldehyde-3-phosphate; Glycerol-3P: glycerol-3-phosphate; Malonyl-ACP: malonyl-CoA acyl carrier protein; TCA: tricarboxylic acid cycle; CIT: citrate; OAA: oxaloacetate; α-KG: α-ketoglutarate

Increasing expression levels of genes involved in fatty acid synthesis can directly enhance intracellular fatty acid synthesis, which in turn provides more precursors for fengycin synthesis. In the fatty acid synthesis pathway, the acetyl CoA is firstly conversed to malonyl CoA via the catalyzation of carboxylase complex (AccD, AccA, AccB, and AccC), and subsequently, FadD (malonyl CoA acyl-carrier protein transacylase) catalyzes the conversion of malonyl CoA to malonyl-ACP (malonyl-CoA acyl carrier protein), after which the synthesis and elongation of fatty acid chains will be started (Fig. 2) [89]. In *B. subtilis*, the conversion of acetyl CoA to malonyl CoA is the rate-limiting step in fatty acid synthesis [89], and high concentrations of malonyl CoA can promote the synthesis of long-chain fatty acids without feedback inhibition [90]. It has been revealed that the overexpression of carboxylase complex genes can significantly enhance fengycin synthesis [46, 74, 75]. Contrary to expectations, Gao et al. [75] reported that the overexpression of *accBC* significantly increased the fengycin production, while the overexpression of *accDA* resulted in a decrease. The overexpression of the biotin ligase coding gene *birA* and the biotin carboxylase II coding gene *yngH*, which are also involved in converting acetyl CoA to malonyl CoA, could also increase fengycin production [46, 48]. However, although *ldeHA*, which encodes biotin carboxylase, has been reported to increase ACC activity and promote fatty acid biosynthesis [91], overexpression of *ldeHA* resulted in a significant decrease in fengycin production [75]. This may be due to the disruption of intracellular balance. Based on the presented results, it can be concluded that an increased expression level of key enzymes may lead to varying trends in different strains due to the different distribution of metabolic fluxes. Therefore, to increase the production of target product, it is necessary to analyze the relevant metabolic pathway and perform a global analysis based on the metabolic fluxes.

In *B. subtilis*, the synthesis of branched-chain fatty acids starts with a reaction of branched-chain α-ketoacyl CoA and malonyl-ACP catalyzed by FabHB (β-keto-acyl carrier protein synthase III) [89], and it has been reported that overexpression of *fabHB* has a positive effect on fengycin production [54, 75]. Fu et al. [76] discovered that the knockout of *lpdv*, which encodes 2-oxoisovalerate dehydrogenase responsible for the degradation of branched-chain amino acids and the synthesis of straight-chain fatty acids, resulted in a marked reduction in fengycin production in *B. subtilis* NCD-2. As lipoic acid synthetase LipA is involved in the synthesis of lipoic acid during fatty acid synthesis, while Lpdv and lipoamide acyltransferase BkdB are involved in the conversion of branched α-ketoacids to branched-chain α-ketoacyl-CoA, overexpression of *lpdv*, *bkdB* and *lipA* can increase the intracellular content of branched-chain α-ketoacyl-CoA, which enhances the supply of precursors for branched-chain fatty acid biosynthesis, and in turn promotes fengycin synthesis [75].

It is worth noting that the process of fatty acid synthesis can be affected by the feedback inhibition of long-chain acyl-CoA [89, 92]. Thus, excessive enhancement of fatty acid synthesis pathway alone may lead to the accumulation of long-chain acyl-CoA, which can inhibit ACC activity and ultimately affect lipopeptide synthesis. Jin et al. [48] revealed that knocking out *fadB*, which impairs the fatty acid β-oxidation pathway, or overexpressing *yhfL*, the gene encoding the fatty acyl-CoA ligase for promoting the formation of fatty acyl-CoA from fatty acids, both enhanced fatty acyl-CoA synthesis and facilitated fengycin synthesis. However, the combination of knocking out *fadB* and overexpressing *yhfL* reduced fengycin yield in *B. subtilis* BSJ022, which may be due to the feedback inhibitory effect of accumulated long-chain acyl-CoA. Previous reports have shown that the thioesterase TesA from *Escherichia coli* exhibits high acyl-ACP and acyl-CoA thioesterase activities, which catalyzes the synthesis of fatty acids and relieves the feedback inhibition of ACC activity by long-chain acyl-CoA [89, 92]. Thus, further overexpressing of *yngH* and *tesA* in *B. subtilis* BSJ022 promoted the fengycin production because of the release of feedback inhibition of ACC activity [48].

Notably, microbial co-culture has also been applied for enhancing the supply of fatty acid. Wei et al. [61] increased the fengycin yield by the co-cultivation of *B. subtilis* CGF26-IV, a fengycin-producing strain, and *Yarrowia lipolytica* YL-21, a C16 fatty acid-producing strain, resulted in a fengycin yield of 2000 mg/L, which is 1.5 times that from pure culture.

#### Blocking other competitive synthetic pathways

Normally, *B. subtilis* can co-produce multiple lipopeptides, which may compete for energy, NADPH, and direct precursors such as fatty acids and amino acids. Therefore, blocking other competitive synthesis pathways has the potential to increase fengycin production. It has been found that the disruption of gene clusters of other lipopeptides, such as the open-reading frames *srfAA*, *srfAB*, *srfAC*, and *srfAD* for surfactin synthesis, can effectively promote the yield of fengycin [75, 78, 93, 94]. In contrast, Yaseen et al. [64] revealed that the knockout of *srfAC* did not significantly affect fengycin production, while the deletion of *srfAA* resulted in a significant decrease. It is hypothesized that the reason for this phenomenon may be due to the inclusion of *comS*, the gene encoding an anti-adaptive protein that protects the regulatory factor ComK from post-translational degradation [95], in the open reading frame of *srfAA*. In a later study, Vahidinasab et al. [67] reintroduced *comS* into the genome after deleting the entire *srfAA-AD* gene cluster in *B. subtilis* BMV12 and BMV13. However, the deletion still significantly reduced plipastatin production in both strains. Previous research has indicated that the *fen* gene cluster does not contain a gene for external thioesterase/acyltransferase [96]. As fengycin is synthesized in a similar way to surfactin, SrfAD may also be involved in fengycin biosynthesis as an acyltransferase [96]. All of these results indicate that the biosynthesis of different lipopeptides is interconnected rather than independent. Therefore, further investigation of the interactions between the biosynthesis of different lipopeptides is essential to provide a basis for the knockout of by-products.

### Effect of regulatory factors on fengycin production

It is notable that the availability of precursors and the distribution of metabolic fluxes are not the sole factors influencing the synthesis of fengycin. Many regulatory factors can influence the production of fengycin by regulating the expression of the key genes. Furthermore, the regulatory factors within the regulatory network interact with each other, potentially resulting in an indirect effect on the synthesis of fengycin.

It has been proved that the 4’-phosphopantetheinyl transferase (PPTase) encoded by *sfp* plays an important role in lipopeptide synthesis [32]. Lipopeptides, including fengycin, are synthesized by giant NRPS, which require post-translational modification from an inactive apo form to an active holo form by PPTase [31, 32, 77, 85]. The analysis of the *B. subtilis* 168 genome revealed the complete NRPS gene cluster responsible for the synthesis of fengycin and surfactin [96, 97], indicating its potential as a producer. However, *B. subtilis* 168 cannot synthesize fengycin or surfactin due to a frameshift mutation in *sfp* [31, 77]. The mutation prevents the translation of *sfp* into a functionally active enzyme, which in turn prevents the activation of NRPS. The introduction of a functional heterologous *sfp* into *B. subtilis* 168 enabled the synthesis of fengycin and surfactin, which directly demonstrates the importance of *sfp* in lipopeptide synthesis [45, 78]. However, when only the *sfp* was introduced into *B. subtilis* 168, the production of fengycin remained low or even undetectable [77], which suggests that factors beyond PPTase may also be important for fengycin synthesis.

**Fig. 3:**
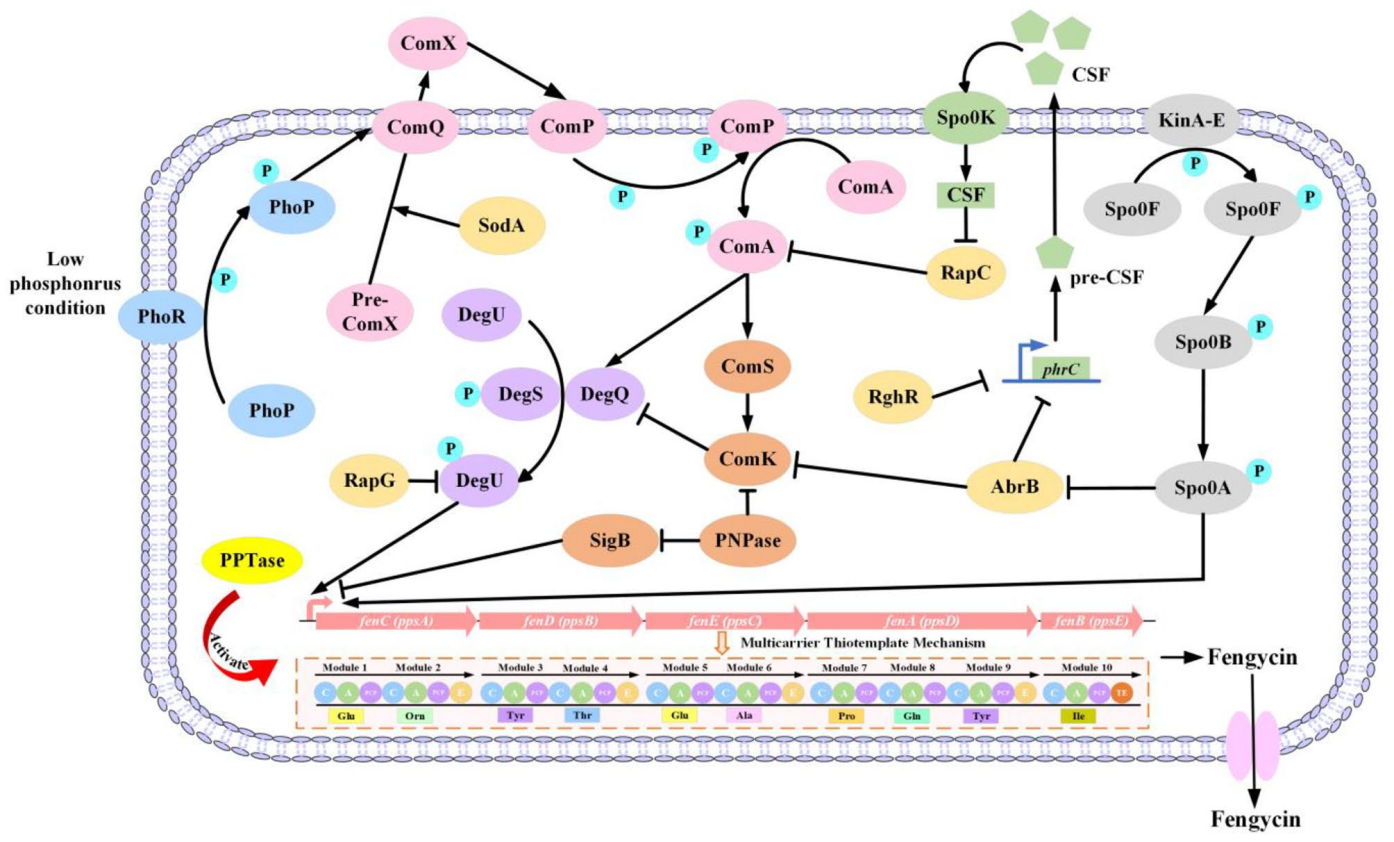
The regulatory network associated with fengycin synthesis. The T-bar represents negative effects and the arrow represents positive effects. The “P” in the circle represents the phosphoryl group

The *comQXPA* gene cluster encodes the quorum-sensing (QS) system of *B. subtilis*. The QS system regulates various processes, including antibiotic synthesis, receptor formation, biofilm formation, and spore production [79]. During the regulation process, signal peptide ComX is modified by isoprenyl transferase ComQ and then secreted outside the cell. When the concentration of ComX reaches a certain threshold with the increase of cell concentration, it prompts the histidine protein kinase ComP to autophosphorylate. Then the phosphoryl group is transferred to response regulator ComA, and the phosphorylated ComA binds to a specific locus to regulate the transcription and expression of corresponding genes (Fig. 3) [79, 95, 98]. It has been shown that the knockout of *comA* in *B. amyloliquefaciens* fmbJ resulted in a significant decrease in fengycin yield, while the overexpression of *comA* led to a 2.6-fold increase [80], indicating that ComA is an important regulator in fengycin synthesis. In addition, Zhou et al. [79] overexpressed *comQXPA* from *B. amyloliquefaciens* HYM-12, resulting in a 35% increase in plipastatin production in the single QS M-24:P_*srfA*_ and a 115% increase in the double QS M-24:P_*srfA*_. This also demonstrated the positive regulation of plipastatin production by QS system.

The ComP-ComA two-component system may regulate fengycin synthesis by activating DegQ. DegQ, a 46-amino acid protein coding for a pleiotropic regulator, can directly stimulate the autophosphorylation of DegS. The activated DegS-P then transfers the phosphoryl group to DegU, and the phosphorylated DegU-P can regulate the expression of related genes [8, 81]. The knockout of *comA* resulted in a significant down-regulation of the expression of *degU* [80], indicating that ComA positively regulates the DegS-DegU two-component regulatory system. It is hypothesized that this regulatory effect may be mediated by DegQ, since ComP-ComA positively regulates the expression of *degQ* [95, 99]. Additionally, previous studies have shown that the yield of fengycin significantly decreased in strains with *degU* knockout [80, 81], and the expression level of *fenA* was also down-regulated [81]. Da-Eun et al. [82] substituted the “A” at position 32 of *degU* with a “T”, resulting in an approximately 2-fold increase in both the transcript level of *pps* gene cluster and their promoter activity, as well as a similar increase in the production of fengycin. This demonstrates that DegU is also a positive regulator for fengycin synthesis.

However, a single-base mutation is present in the −10 box of the promoter of *degQ* in *B. subtilis* 168, resulting in a reduced phosphorylation transfer for DegU activation [8, 100]. The expression of *degQ* increased plipastatin yield by about 10-fold [77]. Similarly, Lilge et al. [8] reported that the expression of *degQ* in *B. subtilis* JABs24 resulted in a six-fold increase in fengycin production, while the deletion of *degQ* in *B. subtilis* DSM10^T^ resulted in a five-fold decrease. In addition, the positive regulatory effect of *degQ* on the expression of fengycin synthase genes has also been proved by quantitative reverse transcription PCR [81, 101]. Surprisingly, the introduction of *sfp* and *degQ* from different sources may lead to different types of plipastatin [102].

In addition to the regulatory factors mentioned above, the polynucleotide phosphorylase (PNPase), encoded by *pnpA*, has also been shown to be involved in the regulation of fengycin synthesis. Yseen et al. [64] demonstrated that although the knockout of *pnpA* increased the expression level of *fen* gene cluster, the fengycin production in mutant strains *B. subtilis* BBG235 and BBG236 decreased significantly to approximately 70% and 40%, respectively. Combined with the results of previous studies, it can be concluded that PNPase plays a dominant role in fengycin synthesis mainly due to its effect on the synthesis of carbon metabolism precursors [64], the expression of regulator coding gene *sigB* [103, 104], the expression of ComS [64], and the expression of regulatory factor ComK [64, 104].

In bacteria, the production of antibiotics and secondary metabolites is also regulated by phosphate, and PhoR/PhoP two-component system is one of the most crucial regulatory systems for *B. subtilis* to adapt to phosphate-limited conditions [76]. In recent years, it has been discovered that the PhoR/PhoP two-component system also affects lipopeptide synthesis. Guo et al. [83] proposed that the inactivation of either *phoR* or *phoP* in *B. subtilis* NCD-2 strain reduced fengycin production, and the regulation of fengycin production by the PhoR/PhoP two-component system occurred only in low-phosphate medium and not in high-phosphate medium. They also demonstrated that *phoP* positively regulates the expression of *fenC* in low-phosphate medium. Subsequent proteomic level analysis revealed that PhoR/PhoP two-component system regulates fengycin synthesis by influencing the expression of proteins and genes related to branched-chain amino acid biosynthesis [76].

Lipopeptide synthesis shares regulatory mechanisms with other starvation-induced activities, such as sporulation and competence formation [105]. During periods of nutrient deprivation or stress, *B. subtilis* enters a state of spore dormancy, and the synthesis and secretion of enzymes or compounds ceases. Thus, spore formation is detrimental to the synthesis of secondary metabolites, and it has been shown that the non-sporulating *B. subtilis* enhances the production of surfactin in high-density fermentations [106]. For fengycin synthesis, knocking out the genes encoding the sporulation pathway-phase proteins SpoIIIE and SpoIVB in *B. subtilis* BSf04-2 increased fengycin production by 37.31% and 12.53%, respectively, which may be due to the up-regulation of glycolytic pathway to increase the utilization of metabolic substrates and the up-regulation of the expression of genes related to the synthesis of branched-chain amino acid and branched-chain fatty acid [75, 106]. Moreover, overexpression of *spo0A*, the gene responsible for spore formation, resulted in a 3.2-fold increase in fengycin production, while a significant decrease in fengycin yield and the expression levels of *comA* and *degU* were observed in the mutant strain with the knockout of *spo0A* [80].

Additionally, the synthesis of fengycin is regulated by genes related to biofilms. Zhou et al. [84] discovered that inactivating of GltB, a regulator associated with biofilm synthesis, not only caused marked defects in biofilm formation in *B. subtilis* Bs916, but also led to a severe decrease in *fen* transcript levels and fengycin production. While Gao et al. [75] showed that the deletion of *tapA-sipW-tasA* and *epsAB*, which are also linked to biofilm formation, could enhance fengycin production without significantly affecting cell growth or glucose consumption. The production of fengycin was not significantly affected by the global regulator CodY [80]. However, the current regulatory network for fengycin synthesis is incomplete, and further systematic and in-depth studies on the regulatory mechanisms of fengycin synthesis are still necessary.

### Application of promoter engineering in fengycin production

Compared with regulation at translational level, which is challenging due to complex spatial structures and regulatory mechanisms, regulation at transcriptional level is more effective. The P_*pps*_ promoter for *pps* gene cluster has limited expression strength and is regulated by a complex inductive regulatory mechanism, which limits the fengycin synthesis. At the same time, the *pps* gene cluster is approximately 37 kb in length, and its excessive length makes it difficult to enhance transcriptional levels by increasing its copy number. Therefore, optimizing the promoter of *pps* gene cluster appears to be a viable solution. The effects of a variety of promoters, including P_*repU*_ [86], P_*amyQ*_ [87], P_*veg*_ [45, 67, 75], P_*ylb*_ [48], P_*fen*_ [48, 85], P_*srfA*_ [48, 79] and MtP_*srfA*_ [79], on fengycin production have been successfully investigated (Table 1). However, only a limited number of promoters have been studied, and further research is still needed to investigate the effects of more promoters.

**Table 1:** The effect of promoter replacement on fengycin production

Strain	Description	Fengycin	Reference
*B. subtilis* BMG01	Native P_*pps*_	91 ± 11.2 mg/L	[86]
*B. subtilis* BMG03	Replacement of P_*ppsA*_ with P_*repU*_	507 ± 6.42 mg/L	
*B. subtilis* Bs2500	Native P_*pps*_	Not detected	[87]
*B. subtilis* Bs2508	Replacement of P_*pps*_ with P_*amyQ*_	434 mg/L	
*B. subtilis* BSUY00	Native P_*pps*_	71.21 mg/L	[45]
*B. subtilis* BSUY04-1	Replacement of P_*ppsA*_ with P_*veg*_	371.63 mg/L	
*B. subtilis* BSf04	Native P_*ppsA*_	2.79 mg/L	[75]
*B. subtilis* BSf04-2	Replacement of P_*ppsA*_ with P_*veg*_	21.94 mg/L	
*B. subtilis* BMV9	Native P_*ppsA*_	15 mg/L	[67]
*B. subtilis* BMV11	Replacement of P_*ppsA*_ with P_*veg*_	70 mg/L	
*B. subtilis* BSJ00	Native P_*pps*_	121.20 mg/L	[48]
*B. subtilis* BSJ02	Replacement of P_*pps*_ with P_*ylb*_	137.05 mg/L	
*B. subtilis* BSJ00	Native P_*pps*_	121.20 mg/L	[48]
*B. subtilis* BSJ03	Replacement of P_*pps*_ with P_*fen* FZB42_	11.53 mg/L	
*B. subtilis* BSJ00	Native P_*pps*_	121.20 mg/L	[48]
*B. subtilis* BSJ01	Replacement of P_*pps*_ with P_*srfA*_	88.18 mg/L	
*B. subtilis* M-24	Native P_*pps*_		[79]
*B. subtilis* M-24:MtP_*srfA*_	Replacement of P_*pps*_ with MtP_*srfA*_	Increased by 350%	
*B. subtilis* M-24	Native P_*pps*_		[79]
*B. subtilis* M-24:P_*srfA*_	Replacement of P_*pps*_ with P_*srfA*_	Increased by 35%	
*B. subtilis* BBG111	Native P_*pps*_		[85]
*B. subtilis* BBG203	Replacement of P_*pps*_ with P_*fen* BBG21_	10-fold increased	

It is important to note that the strength of a promoter can be significantly influenced by culture conditions, such as medium composition [85], temperature and pH [107]. The trend of promoter strength may be reversed under different culture conditions [45]. On the other hand, a high promoter strength does not always lead to increased fengycin production [75]. This may be caused by the gap between transcription and translation levels and the imbalance in intracellular metabolic fluxes caused by a high-intensity promoter. Therefore, although many promoters have been identified through transcriptomic data and related databases and applied to biological system optimization and metabolic engineering, it remains essential to adopt reporter genes and conduct systematic analyses of promoter function based on transcriptomic data analysis to better achieve our goals.

### Application of genome-engineering in fengycin production

Genome shuffling is a highly effective method for rapidly obtaining microbial strains with desirable industrial phenotypes, and one of its advantages is that it can improve strain phenotypes without a clearly defined background of metabolic regulation [108]. The derivative strain F2-72 (FMB72), which was obtained through two rounds of genome shuffling from *B. amyloliquefaciens* ES-2-4, exhibited an 8.30-fold increase in fengycin production, and the transcription level of *fenA* in the FMB72 strain was up-regulated by 12.77 times [20]. Furthermore, comparative proteomic analysis identified 50 proteins with differential expression that are involved in various functions [88]. Among the 44 identified proteins, the mRNA levels of signal proteins ComA and Spo0A were up-regulated by 5.8-fold and 12.1-fold, respectively, which may explain the significant increase in fengycin production in the recombinant strain FMB72.

The metabolic networks of microorganisms are intricate and interconnected. Modifications to specific genes or metabolic pathways can impact the metabolic flux of other pathways. Thus, the overall analysis of metabolic network is essential for achieving the defined goals. The genome-scale metabolic network model (GSMM) based on systems biology can comprehensively and accurately analyze the linkages and variations between metabolic fluxes in microorganisms, which allows for a more precise regulation of the production of target products [109]. Therefore, based on the GSMM with genome sequence annotation and integration of detailed biochemical information for a fengycin-synthesizing *B. subtilis*, He et al. [74] conducted a detailed analysis of the distribution of metabolic fluxes in the ground of fengycin-synthesizing strain, and the key genes that affect fengycin synthesis, including *pnpA*, *accA*, *cypC*, *gapA*, *ppsE*, *hisD*, *phoP* and *yhfT*, were identified by FBA (Flux Balance Analysis) and MOMA (Minimization of Metabolic Adjustment) prediction. Compared with the parent strain, fengycin production was increased by 56.4% with the overexpression of *accA* alone, by 101.9% with the overexpression of *accA* and *cypC*, and by 2.26-fold with the combined overexpression of *accA*, *cypC* and *gapA*. Furthermore, the model-predicted key genes, *pnpA* [64] and *phoP* [83], have also been confirmed to be positive factors for fengycin production. These results demonstrate the reliability and significance of the GSMM.

## Conclusion and prospects

Fengycin has received considerable attention for its potential applications in agriculture, food and medical industries. Therefore, an increasing number of studies have focused on its synthesis and production. This review summarizes the synthesis mechanism of fengycin and strategies for enhancing fengycin production through microbial fermentation.

However, although numerous studies have focused on improving fengycin production, its high cost remains a bottleneck for industrial production. The range of low-cost biomass used for fengycin production remains limited, and there are still many inexpensive biomasses and waste materials that have the potential to serve as fermentation substrates. Further research is needed to develop these options. Besides, the utilization efficiency of these low-cost biomass is low, and the current research on low-cost biomass as a substrate is mainly carried out with its purified products. For example, in the studies regarding the production of fengycin from lignocellulose, fermentations were conducted with xylose or arabinose, which are the main pentoses in hydrolysis products, and the direct utilization of lignocellulosic hydrolysate for fengycin production remains a gap. Therefore, additional research is required. In addition, optimizing the downstream purification process and reducing the treatment costs are also essential to promote the industrial production of fengycin.

The development of chassis cells is also a challenge for the production of fengycin. Currently, there are limited strains available for fengycin synthesis, and many non-model strains not only have low yields but also have difficulties in genetic modification due to the presence of modification restriction systems, which limits the realization of functional diversity and further increases in fengycin yields. Fortunately, the ongoing development of genetic engineering technology and synthetic biology has led to the successful creation of more genetic editing tools, which will aid in the screening and application of more high-yielding, stable and resistant strains in future research.

Modifying the expression levels of regulators involved in fengycin synthesis and enhancing specific pathways, such as the fatty acid synthesis pathway, have been shown to be effective in increasing fengycin production. However, intracellular metabolic pathways are interrelated, and the enhancement of a single pathway may cause excessive perturbation or even imbalance of intracellular metabolic fluxes, which can lead to unintended results. Therefore, the overall analysis of metabolic fluxes and regulatory networks is particularly important. With the development of omics, more regulatory genes and transcription factors have been identified, which will greatly contribute to the further improvement of metabolic and regulatory networks associated with fengycin synthesis and facilitate the exploration of further information.

Furthermore, cyclic lipopeptides exhibit analogous structures and similar synthesis mechanisms. Therefore, the investigation of other cyclic lipopeptides can serve as reference points and sources of inspiration for future studies in fengycin. For example, Li et al. [110] demonstrated that the efflux of surfactin in *B. subtilis* THY-7 was dependent on proton motive force (PMF) rather than ATP hydrolysis, and overexpression of the transporters YcxA、KrsE and YerP increased surfactin production by 89%, 52% and 145%, respectively. These results may serve as a guide for future research on the efflux of fengycin. In addition, genome reduction has already been employed in *B. amyloliquefaciens* LL3, which resulted in an approximately 9.7% increase in surfactin titer [111]. Since the genome-reduced strain exhibited more superior performance in growth rate, transformation efficiency, intracellular reducing power level, and heterologous protein expression capacity, it represents another potential strategy for improving fengycin production.

## Data Availability

No datasets were generated or analysed during the current study.
